# Intrafamily Transmission of HCV Need to More Discussion

**Published:** 2011-02-01

**Authors:** H Lu

**Affiliations:** 1 Department of Infectious Disease, Shanghai Public Health Clinical Center, Fudan University, Caolang Road 2901#, Jinshan District, Shanghai, 201508, P.R China

**Keywords:** Intrafamily transmission, HCV, Hemophilia, Iran

Dear Editor,

Chronic hepatitis C virus (HCV) infection is the major cause of liver disease related to high morbidity and mortality in hemophilic patients who needs regular blood product administration.[1] Although genotype of infecting HCV is one of the prime predictors of response to antiviral therapy,[2] however, its distribution in hemophilic patients is still unclear.[3]

The article titled; Distribution of hepatitis C virus genotypes in Iranian patients with congenital bleeding disorders, revealed the genotype distribution of HCV in Iranian hemophilic patients.[4] In this study, results demonstrate genotype 1a was the most frequent HCV genotype (58%), followed by genotype 3a (18.5%) and genotype 1b (14.7%). Mixed genotypes were also detected in 6.2%. These findings were compatible with other similar studies on HCV infected patients in Iran. In USA and Western Europe, HCV genotypes 1a and 3 are more predominant, 1b and 2 in South Europe and genotype 4 in Africa and Middle East.[5] This pattern of HCV genotypes in Iranian hemophilic patients is similar to which of Western Europe and different to which of neighbor countries.

This work has enriched the data of genotype distribution in different areas. Nevertheless, genotype is an important parameter used in selecting an antiviral therapy and genotyping and subtyping of HCV is relevant to the epidemiology of HCV, I believe this article may give some suggestive help in clinical management against chronic HCV infection.
